# Identification of Druggable Genes for Asthma by Integrated Genomic Network Analysis

**DOI:** 10.3390/biomedicines10010113

**Published:** 2022-01-06

**Authors:** Wirawan Adikusuma, Wan-Hsuan Chou, Min-Rou Lin, Jafit Ting, Lalu Muhammad Irham, Dyah Aryani Perwitasari, Wei-Pin Chang, Wei-Chiao Chang

**Affiliations:** 1 Department of Clinical Pharmacy, School of Pharmacy, Taipei Medical University, Taipei 11031, Taiwan; adikusuma28@gmail.com (W.A.); ocean.chou@tmu.edu.tw (W.-H.C.); jennlin@tmu.edu.tw (M.-R.L.); jkt5265@tmu.edu.tw (J.T.); 2 Department of Pharmacy, Faculty of Health Science, University of Muhammadiyah Mataram, Mataram 83127, Indonesia; 3 Faculty of Pharmacy, University of Ahmad Dahlan, Yogyakarta 55164, Indonesia; lalu.irham@pharm.uad.ac.id (L.M.I.); dyah.perwitasari@pharm.uad.ac.id (D.A.P.); 4 School of Health Care Administration, College of Management, Taipei Medical University, Taipei 11031, Taiwan; 5 TMU Research Center of Cancer Translational Medicine, Taipei 11031, Taiwan; 6 Department of Pharmacy, Wan Fang Hospital, Taipei Medical University, Taipei 11696, Taiwan; 7 Integrative Research Center for Critical Care, Wan Fang Hospital, Taipei Medical University, Taipei 11696, Taiwan; 8 Department of Pharmacology, National Defense Medical Center, Taipei 11490, Taiwan

**Keywords:** asthma, bioinformatic, drug repositioning, genome-wide association study, phenome-wide association study

## Abstract

Asthma is a common and heterogeneous disease characterized by chronic airway inflammation. Currently, the two main types of asthma medicines are inhaled corticosteroids and long-acting β2-adrenoceptor agonists (LABAs). In addition, biological drugs provide another therapeutic option, especially for patients with severe asthma. However, these drugs were less effective in preventing severe asthma exacerbation, and other drug options are still limited. Herein, we extracted asthma-associated single nucleotide polymorphisms (SNPs) from the genome-wide association studies (GWAS) and phenome-wide association studies (PheWAS) catalog and prioritized candidate genes through five functional annotations. Genes enriched in more than two categories were defined as “biological asthma risk genes.” Then, DrugBank was used to match target genes with FDA-approved medications and identify candidate drugs for asthma. We discovered 139 biological asthma risk genes and identified 64 drugs targeting 22 of these genes. Seven of them were approved for asthma, including reslizumab, mepolizumab, theophylline, dyphylline, aminophylline, oxtriphylline, and enprofylline. We also found 17 drugs with clinical or preclinical evidence in treating asthma. In addition, eleven of the 40 candidate drugs were further identified as promising asthma therapy. Noteworthy, *IL6R* is considered a target for asthma drug repurposing based on its high target scores. Through in silico drug repurposing approach, we identified sarilumab and satralizumab as the most promising drug for asthma treatment.

## 1. Introduction

Asthma is a prevalent chronic respiratory disease that can adversely influence patients’ quality of life of all ages and genders. Patients usually suffer from repeated episodes of wheezing, shortness of breath, tightness of the chest, and coughing [1,2]. Asthma, a heterogeneous disease, is classified into different clinical phenotypes such as allergic asthma, non-allergic asthma, adult-onset asthma, asthma with persistent airflow limitation, and asthma with obesity [3]. It is estimated that approximately 339 million people worldwide have asthma, with an estimated prevalence rate of 1–18%. In addition to the rising prevalence, morbidity and mortality rates have increased over the past few decades [1,4,5]. The increasing number of asthma patients is a burden to medical investments and represents increased care costs for families and communities [5]. The combination of genetic and environmental factors is widely thought to play a crucial role in the pathogenesis and treatment efficacy of asthma [6,7]. Studies of gene–environmental interactions may help elucidate disease mechanisms and classify particular genes or exposures in the same pathway [8].

The main goal of asthma treatments is to control the symptoms properly and prevent exacerbation. Two drugs are generally the leading choices for treating asthma: inhaled corticosteroids and long-acting β_2_-adrenoceptor agonists (LABAs). Severe asthma patients need add-on therapies, such as biological drugs (Monoclonal antibodies) that target specific molecular pathways [3,9]. However, these drugs do not prevent severe disease exacerbation in a significant proportion of patients [10]. This indicates a need to develop novel anti-asthma therapies. The development of new small-molecule drugs is a risky undertaking that costs hundreds of millions of dollars and needs years of research and clinical trials. Unfortunately, many drugs failed during safety testing in Phase I trials and were not able to move forward in the clinical trial process [11]. With the rapid development of bioinformatics knowledge and big biological data, drug repositioning strategies can be used to seek new indications for approved drugs [11,12,13]. It can significantly reduce the time, costs, and risks of the drug development process [14,15], and serves as a solution for the pharmaceutical industry to improve their profits and overcome future medical challenges.

New drug discovery is a challenging process. Several methods can be used to find new indications for existing drugs, such as knowledge-based, activity-based, and in silico-based drug repurposing [16]. In the present study, we performed in silico drug repurposing with the integration of both gene network and bioinformatic analytic approaches to find promising candidate drugs for asthma therapy.

## 2. Materials and Methods

### 2.1. Study Design

**Figure 1:**
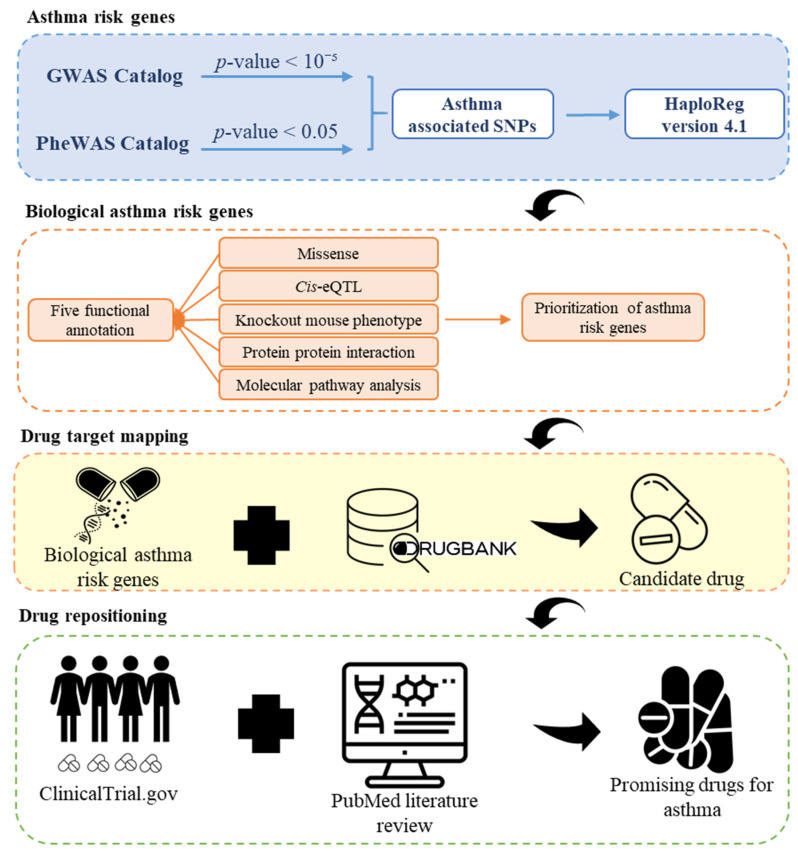
Study design of the drug repurposing approach to identify promising drugs for asthma. Asthma-associated SNPs were identified through GWAS and PheWAS Catalog. Next, the asthma-associated SNPs were extended by HaploReg v4.1 to identify asthma risk genes. Five criteria of functional annotation were used to prioritize candidate genes. Candidate genes were linked to drugs through the DrugBank database. Furthermore, we used ClinicalTrial.gov and PubMed literature review to find a promising repurposed drug for asthma.

An overview of the research design is shown in Figure 1. We acquired asthma-associated single-nucleotide polymorphisms (SNPs) through genome-wide association studies (GWASs) [17] and Phenome-wide association studies (PheWAS) Catalog [18] on 8 May 8 2019. GWAS catalog is one of the largest resources available online (https://www.ebi.ac.uk/gwas/, 8 May 2019). The purpose of GWAS is to decipher associations between common genetic variants and diseases or traits. In the “opposite” orientation of GWAS, PheWAS investigates the correlation between diseases or traits and particular genetic variants [19]. Next, HaploReg v4.1 (Massachusetts Institute of Technology, 77 Massachusetts Avenue, Cambridge, MA, USA) [20] extended the asthma-associated SNPs to identify asthma risk genes with criterion *r*^2^ > 0.8 in the Asian population. Five functional annotation criteria have been used to give priority to these genes. We prioritized genes as “biological asthma risk genes” if they were annotated to meet more than or equal to two criteria (score ≥ 2). Then, we mapped biological asthma risk genes based on DrugBank [21] to find candidate drugs. ClinicalTrial.gov (https://clinicaltrials.gov/) and PubMed literature reviews accessed on 26 October 2021 were used to identify the most promising drug for asthma.

### 2.2. Biological Asthma Risk Genes

Biological asthma risk genes were obtained based on the five functional annotations that met two or more of the requirements (i.e., had a score of ≥ 2). We adopted the scoring system from a previous study by Okada et al., which predicted the candidate drugs for rheumatoid arthritis [22]. Each gene in the risk loci for asthma was scored according to the following five criteria: (1) Missense: genes containing asthma risk SNPs with linkage disequilibrium (*r*^2^ > 0.80) and were annotated as missense mutations in HaploReg v4.1; (2) Cis-expression quantitative trait locus (*Cis*-eQTL): genes containing asthma risk SNPs with significant *cis*-eQTL effect in the lung; (3) Knockout mouse phenotype (KO mice): genes with a significance of False Discovery Rate (FDR) *q* < 0.05 in the over-representation analysis (ORA) using Mammalian Phenotype Ontology (MP) from WebGestalt (2019) [23]; (4) Protein–protein interactions (PPIs): genes were prioritized by biological process Gene Ontology (GO) categories in WebGestalt 2019 [23]. Those with an FDR *q* < 0.05 were considered significant, and (5) Molecular pathway: genes were prioritized using the Kyoto Encyclopedia of Genes and Genomes (KEGG), an online biochemical route database from WebGestalt 2019 [23]. Genes involved in significantly enriched pathways (FDR *q* < 0.05) were assigned one point. Each gene was scored based on the number of matched criteria (scores ranged from 0 to 5 for each gene).

### 2.3. Drug Mining and Prioritization

In this step, we mapped biological asthma risk genes to DrugBank (data released on 3 January 2021) to find candidate drugs for asthma. DrugBank is an online database that provides information about drugs and gene targets as a bioinformatics and cheminformatics resource for drug discovery in clinical medicine communities. The DrugBank can be used for in silico drug target discovery, drug design, drug docking or screening, drug metabolism prediction, drug interaction prediction, and general pharmaceutical education [21]. Several parameters were used to query the databases, such as drugs with pharmacological activity, human effectiveness, approved annotations, clinical trials, or experimental drugs. Furthermore, all drugs were confirmed by ClinicalTrial.gov (https://clinicaltrials.gov/; accessed on 26 October 2021) to verify if each drug is under clinical investigation for asthma or other diseases.

### 2.4. Statistical Analysis

In this study, all analytic workflows were performed on RStudio version 4.0.3 (RStudio, 250 Northern Ave, Boston, MA 02210). Missense and Cis-expression quantitative trait locus (*Cis*-eQTL) were performed in R using the haploR package [24]. Over-representation analysis (ORA), including Knockout Mouse Phenotype, PPI network, and Molecular Pathway, were performed using the WebGestalt R package [25].

## 3. Results

### 3.1. Identification of Asthma Associated SNP

A total of 969 asthma-associated SNPs were extracted, including 658 from the GWAS catalog (*p*-value < 10^−5^) and 336 from the PheWAS catalog (*p*-value < 0.05) (Table S1). Subsequently, based on the characteristic of *r*^2^ > 0.8 used in Asian populations, we extended the number of SNPs by HaploReg v4.1 and obtained 1047 asthma risk genes (Table S2).

### 3.2. Gene-Based Prioritization from Functional Annotation

**Figure 2:**
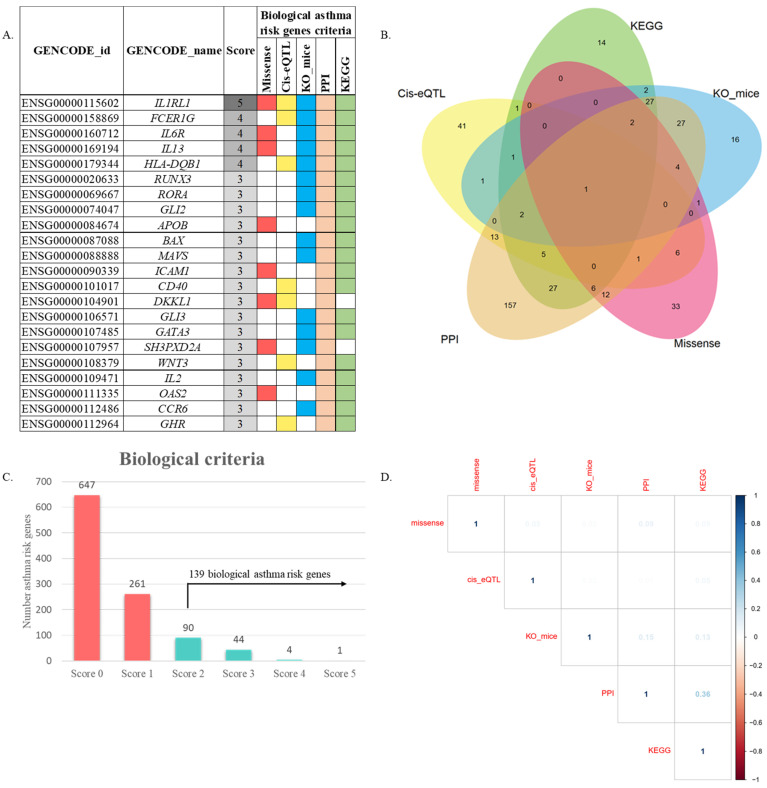
Prioritization of biological candidate gene from asthma risk loci. (**A**) Summary scores derived from 5 criteria are shown. The boxes are filled with different colors to distinguish each functional annotation. Filled boxes indicate fulfilled criteria. Gene with a score ≥ 2 was defined as “biological asthma risk genes.” For complete information, see Table S3. (**B**) Venn diagram shows the prioritization criteria of the biological candidate gene from asthma risk loci. (**C**) Histogram distribution of gene scores. The figure shows 139 genes with total scores ≥ 2. (**D**) Correlogram indicates the pairwise Phi correlation coefficient between the five criteria. The blue color denotes a positive correlation, while the red color denotes a negative correlation.

We utilized five functional annotations to determine which genes have priority for drug discovery. The scoring results were as follow: (1) genes include asthma risk missense variant (*n* = 66); (2) genes with *Cis*-eQTL effect (*n* = 72); (3) genes prioritized by KO mice (*n* = 84); (4) genes prioritized by PPIs (*n* = 284); and (5) genes prioritized by a molecular pathway (*n* = 88). Finally, a total of 139 biological asthma risk gene fulfilled the criteria with a score ≥ 2. We evaluated the gene scores to provide empirical evidence of the pipeline. Our result showed that the top five genes have a score higher than 3, including *IL1RL1*, *FCER1G*, *IL6R*, *IL13*, and *HLA-DQB1* (Figure 2A; Table S3). The distribution score of each criterion is shown in Figure 2B,C. Furthermore, we found that these five criteria showed a low positive correlation (*r* < 0.4) to each other (Figure 2D).

### 3.3. Integrative Analysis for Drug Repositioning

**Figure 3:**
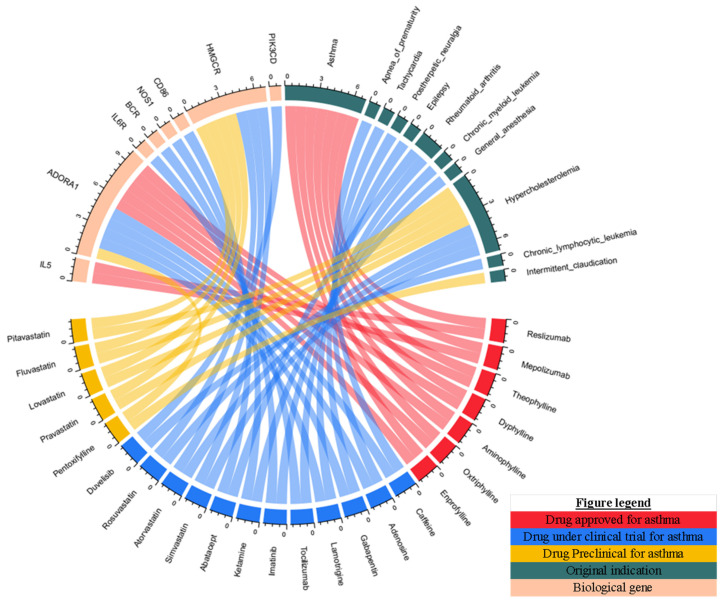
Chord diagram of the connections among biological gene, drug target, and indications identified by clinical trial and PubMed literature review.

**Figure 4:**
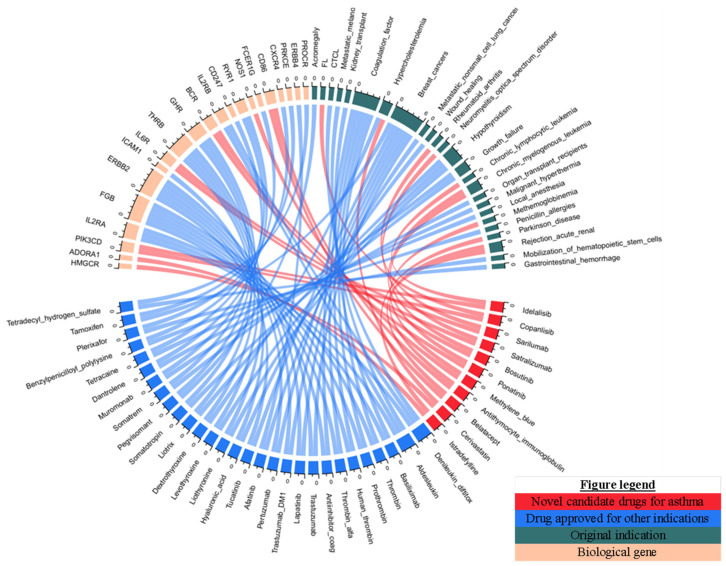
Chord diagram of the connections between biological genes with promising anti-asthma drugs. Connections with biological genes investigated in clinical and preclinical evidence are highlighted in red color.

To identify the candidate drugs for asthma, we mapped biological asthma risk genes to drugs in DrugBank. We evaluated whether genes from the biologically risky gene profiles were pharmacologically therapeutic targets of approved drugs. A total of 64 drugs were identified, which targeted 22 asthma risk genes. Thus, these drugs are considered as candidate drugs for asthma therapy (Table S4). Among these 64 drugs, seven were approved for asthma; twelve were under clinical investigation for asthma; five were supported by preclinical in vivo or in vitro asthma models, and 40 were novel drugs that have not been reported to treat asthma. First, we focused on drugs supported by FDA approval, clinical studies, and preclinical evidence. A total of eight genes were targeted by these drugs, including *IL5*, *HMGCR*, *PIK3CD*, *CD86*, *BCR*, *NOS1*, *IL6R*, and *ADORA1* (Figure 3). Besides, a list of 17 drugs with clinical and preclinical data is shown in Table 1. Thus, these genes are considered promising targets for asthma. Finally, by matching these eight genes to the 40 novel drugs, we identified eleven drugs (idelalisib, copanlisib, cerivastatin, sarilumab, satralizumab, bosutinib, ponatinib, methylene blue, antithymocyte immunoglobulin (rabbit), and istradefylline) matching with seven genes that might be repurposed for asthma (Figure 4).

## 4. Discussion

In this study, GWAS and PheWAS databases were used to extract information from asthma risk loci that could guide the drug repurposing process. One hundred thirty-nine genes were classified as biological asthma risk genes using five biological criteria. Through DrugBank, we identified 64 drugs targeting 22 genes, of which seven drugs were approved for asthma, such as mepolizumab, reslizumab, theophylline, dyphylline, aminophylline, oxtriphylline, and enprofylline. Besides, 17 drugs with clinical and preclinical evidence were found useful for asthma therapy. Therefore, our result suggests that the combination of GWAS, PheWAS, and in silico approach is valuable to provide scientific evidence for asthma drug discovery.

Among the seven promising target genes for asthma, we screened out *IL6R* as a highly promising target for treatment of asthma since the gene also acquired a high systemic score in functional annotations. The role of *IL6R* in asthma has been supported by preclinical and clinical trial evidence. *IL-6* signaling was implied by an animal, genetic association, and clinical studies in allergic asthma. *IL-6* trans-signaling has a pathogenic role in asthma severity-related airways. The *IL-6* trans-signaling large subset was overrepresented by frequent exacerbations, blood eosinophilia, and submucosal T cell and macrophage infiltration [26]. When *IL-6* binds to the *IL-6R*, it does not induce a signaling cascade. However, it is associated with the gp130 signal transducer protein, which triggers the activation of the specific members of the *JAK* family of tyrosine kinase (*JAK1*, *JAK2*, *JAK3*, and *Tyk2*), leading to phosphorylation and activation of the major transcriptional factor controlled by *IL-6* and *STAT3* [27,28]. Furthermore, the secretion of *IL-6* also induced the *C/EBPβ* transcription factor through the *MAPK* pathway [29]. Recent mouse studies, along with the surprising GWAS results demonstrating a genetic link between *IL-6R* and human asthma, indicated that *IL-6* (or *IL-6R*) is a priority for asthma treatment [30,31]. Anti-IL-6R mAb that blocks both mIL-6R and sIL-6R involves activating IL-6 trans-signaling to prevent allergen-induced asthma exacerbations [32,33]. This study found three anti-IL6R mAb, including tocilizumab, sarilumab, and satralizumab. One of these drugs (tocilizumab) has been in clinical trials for asthma (Trial registered in the Australian New Zealand Clinical Trials Registry, number ACTRN12614000123640). By targeting *IL6R*, sarilumab and satralizumab could become novel candidate drugs for asthma treatment options.

Among the target identified, the roles of *HMGCR* and *ADORA1* as implicated in asthma are supported by clinical and preclinical evidence. *HMGCR* plays an essential role in cholesterol and isoprenoids biosynthesis in the mevalonate (MA) pathway. Isoprenoid was associated with asthma-related processes and respiratory disorders, including allergic eosinophilic inflammation [34,35,36,37]. Allergic asthma significantly increased the *HMGCR* expression in the liver, and increased *HMGCR* expression indicates increased cholesterol biosynthesis [38]. Statin drugs, which have been used clinically for hyperlipidemia and cardiovascular diseases for decades, directly inhibit *HMGCR* [35]. In this study, we identified eight statin drugs targeting *HMGCR*. Among them, three drugs (simvastatin, NCT0126643; atorvastatin, NCT00126048; rosuvastatin, NCT00463827) are under clinical trial investigation; four drugs (pitavastatin [39], pravastatin [40], lovastatin [41], and fluvastatin [42]) are supported by in vivo or in vitro preclinical data for asthma. Since several in vivo or in vitro studies showed that statin drugs could reduce inflammatory airways; cerivastatin may become a promising candidate for asthma treatment by targeting *HMGCR* [43,44]. The adenosine receptors (*ADORA1*, *ADORA2A*, *ADORA2B*, and *ADORA3*) have a promising therapeutic role in asthma and chronic obstructive pulmonary disease (COPD) [45]. By activating antagonists *ADORA1/ADORA2B*, adenosine mediates bronchoconstriction and mucin formation and increases endothelial cell permeability in preclinical studies [46]. Several drugs that target *ADORA1* were in preclinical or clinical trials for asthma, as shown in Table 1. In particular, we identified istradefylline as a novel candidate drug for asthma.

Additionally, our bioinformatic networking analysis also showed that *CD86* and *NOS1* are promising targets for asthma drug repurposing. *CD86* is associated with three drugs (abatacept, belatacept, and antithymocyte immunoglobulin (rabbit)). One of these drugs (abatacept) has completed clinical trial phase II (NCT00784459). The expression of *CD86* in B cells was significantly increased in asthma patients. Blocking *CD86* effectively inhibits allergic reactions by decreasing Th2 cytokines production [47,48]. Furthermore, a previous study showed that *NOS1* plays a significant role in asthmatic children [49]. Several drugs were linked to *NOS1*, such as ketamine and methylene blue. In clinical trial phase III, ketamine was used as adjuvant therapy in pediatric emergency patients with acute asthma (NCT03338205). The last category of drugs identified in this study were anticancer drugs (bosutinib, ponatinib, idelalisib, and copanlisib), targeting *BCR* and *PIK3CD*. Herein, *BCR* is linked to three drugs such as imatinib, bosutinib, and ponatinib. Among these drugs, only imatinib is under clinical trial for asthma. In a phase II randomized clinical trial for severe refractory asthma, imatinib could reduce hyperresponsiveness to the airway, mast-cell counts, and tryptase release (NCT01097694). Meanwhile, *PIK3CD* is mapped to three (duvelisib, idelalisib, and copanlisib) types of cancer drugs. Duvelisib is one of the three drugs that have been in a clinical trial for asthma (NCT01653756) and shows good tolerance as reported for each dose variation evaluated [50]. However, anticancer drugs target fast growing cells in the body without considering that the body also contains rapidly dividing non-cancerous cells. These drugs have a narrow therapeutic index, meaning that the doses required to create an anticancer effect and the levels required to produce harmful consequences are similar [51]. Hence, our study did not consider anticancer drugs for asthma drug repurposing due to the high adverse effects.

Drug repurposing offers various advantages over developing an entirely new drug for a given indication, such as fewer risks, lower cost, and shorter development time [52]. Nevertheless, the approach does not always succeed; one example was a recent case of risankizumab in severe asthma. A phase 2a, placebo-controlled trial (NCT02443298) showed that the repurposed drug might not benefit severe asthma patients [53]. The failure may be due to biological variations of biomarkers, etiology of disease, and clinical phenotypes among patients. The deviations lead to different patterns of treatment response. In this study, we included previous reports of any types of asthma (e.g., chronic obstructive asthma, adult-onset asthma, atopic asthma, childhood-onset asthma), and extracted the asthma-associate genetic variants from GWAS and PheWAS catalogs. Without the further specification of patient subgroups, studies on animal models with different phenotypes and clinical trials are necessary to validate the effectiveness of the candidate drugs in practical usage. This study utilized incorporated genetic data, computational methods, and publicly accessible big data sets to prioritize the best candidate genes and identify new drugs for asthma therapy. However, there are some limitations. First, genes from the GWAS and PheWAS catalogs are not always druggable, and not all gene targets emerge distinct pharmacological activity. Our analysis showed that among 139 biological asthma risk genes, however, only 22 genes are druggable. Second, the therapeutic drugs found in this pipeline have not been validated. Further functional studies and clinical studies are required to determine the possibility of clinical application and implementation in our findings.

## 5. Conclusions

Drug repositioning through in silico methods provides a faster drug discovery process to find novel indications for approved drugs in complex human diseases. Through our pipeline, we identified seven approved drugs, 17 drugs with clinical and preclinical evidence, and 40 novel drugs for asthma. In particular, seven gene targets (*HMGCR*, *ADORA1*, *IL6R*, *CD86*, *BCR*, *PIK3CD*, and *NOS1*) are prioritized for asthma drug repurposing. These genes were mapped to eleven novel drugs, which is worthy of further investigation. Among these targets, we highly recommend drugs targeting *IL6R* for asthma repurposing, since the gene had a high systemic score in functional annotations. In summary, our results revealed that the anti-IL6R (Sarilumab and satralizumab) are promising candidates for drug repositioning to asthma therapy. However, it will be helpful to carry out more studies from animal models and clinical trials to determine the mechanisms of anti-IL6R in asthma.

## Figures and Tables

**Table 1:** Asthma candidate drugs supported by clinical trials and preclinical evidence.

Drug Candidate	Gene Target	Drug Action	Current Drug Indication	Phase of Development	N.C.T. Number/PubMed ID
Gabapentin	*ADORA1*	Agonist	Postherpetic neuralgia	Phase IV	NCT00153283
Lamotrigine	*ADORA1*	Inhibitor	Epilepsy	Phase IV	NCT00153244
Simvastatin	*HMGCR*	Inhibitor	Hypercholesterolemia	Phase III	NCT01266434
Ketamine	*NOS1*	Inhibitor	General anaesthesia	Phase III	NCT03338205
Atorvastatin	*HMGCR*	Inhibitor	Hypercholesterolemia	Phases II/III	NCT00126048
Imatinib	*BCR*	Inhibitor	Chronic myelogenous leukaemia	Phase II	NCT01097694
Abatacept	*CD86*	Antagonist	Rheumatoid arthritis	Phase II	NCT00784459
Duvelisib	*PIK3CB*	Inhibitor	Small lymphocytic lymphoma	Phase II	NCT01653756
Adenosine	*ADORA1*	Agonist	Tachycardia	Phase II	NCT01006655
Rosuvastatin	*HMGCR*	Inhibitor	Hypercholesterolemia	Phase I	NCT01411111
Tocilizumab	*IL6R*	Inhibitor	Rheumatoid arthritis	Phases I/II	ACTRN12614000123640, 25930193, 30885880
Caffein	*ADORA1*	Inhibitor	Apnea of prematurity	NA	NCT01057875
Pentoxifylline *	*ADORA1*		Intermittent claudication	-	19905913
Pitavastatin *	*HMGCR*	Inhibitor	Hypercholesterolemia	-	28729731
Pravastatin *	*HMGCR*	Inhibitor	Hypercholesterolemia	-	18835962
Lovastatin *	*HMGCR*	Inhibitor	Hypercholesterolemia	-	25374755
Fluvastatin *	*HMGCR*	Inhibitor	Hypercholesterolemia	-	16630152

* Represents preclinical in vivo or in vitro; NA, not available.

## Data Availability

The data presented in this study are available in supplementary material.

## Supplementary Materials

The following supporting information can be downloaded at: https://www.mdpi.com/article/10.3390/biomedicines10010113/s1, Table S1: Summary of asthma-associated SNPs from GWAS and PheWAS catalogs, Table S2: Summary of 1047 asthma risk genes based on linkage disequilibrium (*r*^2^ > 0.8), Table S3: Summary of 139 biological asthma risk genes, Table S4: Summary of 64 candidate drugs from DrugBank database.
